# Practical and Ethical Solutions for Remote Applied Learning Experiences in Global Health

**DOI:** 10.5334/aogh.2999

**Published:** 2020-08-19

**Authors:** Anna Kalbarczyk, Meagan Harrison, Maria Cecilia Dedios Sanguineti, Juddy Wachira, Carlos A. Faerron Guzman, Bhakti Hansoti

**Affiliations:** 1 Johns Hopkins University, Johns Hopkins Center for Global Health, Baltimore, MD, US; 2 Universidad de los Andes, School of Government, Bogota, CO; 3 Department of Mental Health and Behavioral Sciences, School of Medicine, Moi University, Eldoret, KE; 4 Department of Media Studies, School of Literature, Language and Media, University of Witwatersrand, Johannesburg, ZA; 5 InterAmerican Center for Global Health, CR

## Abstract

Global health trainees rely on immersive experiences to apply their classroom knowledge in real-world settings. However, during the COVID-19 pandemic travel has come to a halt and short-term experiences are no longer available in their current form. As with didactic material, global health programs have an opportunity to innovate the delivery of applied learning, providing trainees with robust, mentored experiences that promote the acquisition of core global health competencies. We provide a series of practical solutions for remote applied learning including case-based learning, pathfinder pedagogy, virtual reality simulations, and twinning. We further describe the role of these approaches in addressing common criticisms of short-term experiences and their potential for creating new win-win dynamics between institutions and trainees.

## Introduction

Short term experiences in global health (STEGHs) are an integral part of many academic programs [1]. Global health trainees from a variety of disciplines (e.g. public health, nursing, medicine, engineering, business, and international relations) rely on in-person immersive international experiences to apply their knowledge, build networks, challenge preconceptions, and gain insights into how the cultural, health systems and resource nuances impact disease epidemiology and health service delivery. STEGHs are not feasible in their traditional form during the COVID-19 pandemic, as international borders have closed. US-based airlines have reduced international flying capacity by over 80–90% percent [2], and universities have banned non-essential travel [3,4,5]; students are grounded.

The COVID-19 pandemic forced academic institutions to task shift from traditional classroom-based teaching strategies and in-person didactics to remote asynchronous approaches. To support this effort, institutions have quickly mobilized strategies to deliver online-only learning resources [6]. Instructors rapidly learned new tools to synchronously and asynchronously deliver material, facilitate student engagement, and assess learning. Digital companies responded by deploying new servers, increasing infrastructure, and offering free access to their services [7]. Some argue that online learning is here to stay, at least for those with access to the technology [7]. Many also believe that the Netflix era of learning promises increased access, variety of educators, and more open-access resources to reach wider audiences [8,9].

Less is understood about how the lack of field training and exposure to different international contexts will impact the training of global health students. For those who conduct practice and research in global health, many questions remain on how to best proceed during future academic years. In this paper we seek to explore alternative applied practicum experiences and provide solutions to educators supporting students in global health.

## Role of Global Health Electives in Training

STEGHs are characterized by the travel of students from high-income countries to low-and middle-income countries (LMICs) to gain work experience, volunteer, teach, or conduct research [10]. These experiences can vary widely in terms of length, location, and scope of work, but at the core of the STEGH is immersion in an international setting [11]. Here we define immersion as experiences that enable learners to have direct, prolonged in vivo contact while immersed in a culture different than their own [12]. There is extensive literature on the specific competencies developed during international experiences depending on the specific trainee population [13]. Several frameworks exist and demonstrate how these competencies support training and development across multi-disciplinary fields [14].

During immersive field experiences, students are expected to gain knowledge about global public health conditions and social determinants of health and work directly with local collaborators, students, field teams, and stakeholders. This gives the trainees an opportunity to implement and contextualize concepts they have learned in classes and see how those concepts play out in reality [15]. Immersion in a different region or cultural context prepares students to be more effective in their careers by helping to develop a better understanding of the unique needs and assets of different communities in LMICs, beyond what they were able to learn from a lecture in class [16]. As a result of their experiences, students who have participated in cultural immersions report increased cultural awareness and sensitivity [17], and cultural immersion has been found to improve cultural competence for students in helping professions such as social work and counseling [18,19]. Among medical students participating in immersive global health experiences, benefits such as increased cultural sensitivity, enhanced community, social, and public health awareness, enhanced clinical and communication skills, more appropriate resource utilization, and a greater understanding of the challenges of working in areas with scarce resources were achieved after their field experience and were sustained one and two years later [20]. With proper guidance, the very nature of global health experiential learning as a cross-cultural experience and the epistemic and normative disruption generated in the student can be a catalyst for transformative change.

Immersive experiences also expose students to different situations, requiring them to adapt. This can present both challenges and learning opportunities for students. Students may learn to adapt to a working environment with limited internet access, diagnostic tests, healthcare infrastructure and amenities [20]. Understanding the nuances of resource limitations allows for innovative solution focused problem solving that can be applied at home and abroad. Students may also be challenged to recognize the many assets of LMICs [21], including resource optimization, resilience, equity-driven healthcare, and community cohesion.

Given the current restrictions on travel due to COVID-19, we expect that international field experiences (both clinical and research centered) for global health students will be postponed, greatly altered, or cancelled altogether. Furthermore, significant concerns have been raised about student safety, program feasibility, and ethical challenges for study abroad programs moving forward [22]. What, then, does this mean for global health education programs that rely on international STEGHs as part of their training, educational offerings, and degree requirements?

Without an international field experience, we must consider whether students will be able to attain and demonstrate the core competencies [23] associated with global health training. This begs the question: is it possible to innovate ways to introduce students who are completing remote applied learning experiences to the personal learnings that come with an immersive experience? And if so, what are the ways in which we can address the important criticisms of STEGHs and make these experiences beneficial for both students and the in-country partners with whom they work?

## Practical Solutions for Applied Practicums

We believe that with innovations using virtual technology, institutions can facilitate remote applied learning experiences in place of immersion experiences that both allow students to practice their global health competencies while also benefiting collaborating partners in the field. We argue that inherent in this unprecedented situation are significant opportunities for innovation that create a new win-win dynamic for both students and international collaborators while addressing significant ethical concerns inherent in many STEGH offerings. Many core competencies achieved through STEGHs remain relevant and achievable, even from a distance. It is incumbent upon academic institutions to continue to provide applied learning experiences to global health trainees; in fact, we view this as an opportunity for innovation in ‘field’ training.

### Strategies to Strengthen Practicum Design

When designing remote applied learning programs, it is important to maintain certain foundational activities that are characteristic of immersive applied learning programs. Even though the physical immersion aspects of a program may not be possible, there are activities in the preparatory, practicum, and post-practicum phases [10] of the learning program experience that can be preserved. We believe there exist innovative pedagogical solutions that can be incorporated or enhanced to achieve this goal.

1. Case-based simulation learning has been shown to encourage acquisition of knowledge, skills, and attitudes by placing events in contexts that promote authentic learning [24], and has been increasingly called for in global health [25,26,27]. By engaging with real-world case studies, we can expose students to stories and situations that they might encounter during an in-country global health immersion experience. In a virtual format, students could potentially benefit from case-study simulations set in multiple different locations, resulting in exposure to and understanding of a broader range of countries, cultures, and contexts.
2. Pathfinder tools essentially function as a “choose your own adventure,” and provide a non-linear narrative where students choose a “path” to follow to gain clues, or experience meant to develop their understanding of a topic. This is a game-based pedagogy that allows for active participation [28]. Using the pathfinder tool, educators can simulate situations in which students interact and make decisions and see results of those decisions in real-time.
3. Virtual Reality: Both case-based learning and pathfinder pedagogies can be augmented when implemented within the context of a virtual reality (VR) experience. VR is being increasingly used in the fields of education and training, and it has been called the “learning aid of the 21^st^ century [29].” There are different levels of immersion that VR can create. Spatial immersion is a perception of being “physically present in a non-physical world” achieved by “surrounding the user of the VR system with images, sound or other stimuli” that feel authentic to the user and provide an “absorbing environment [30].” A combination of different multimedia approaches such as 360 video, interviews and technical guidance from local experts, and virtual “tours” can create an experience in which trainees can be immersed at different levels to comprehensively explore unfamiliar settings and cultural environments, supporting engagement and cognitive empathy [29]. VR glasses or other versions of Head Mounted Displays (HMD) used with headphones are able to produce the visceral feeling of being in the simulated space [30] such as a bustling urban market or a remote village that a global health student might visit in person given the opportunity. This novel approach allows students to observe and step into a role as if they were physically present in the scene. HMDs are expensive, thus cost would be a barrier to participation for this level of immersion; however there are lower-cost mobile and desktop VR technologies that can still provide an effective and engaging experience [31]. While immersion experts maintain that in order to truly believe that one is in a virtual world, all five senses should be involved, most VR environments today focus on sight, “held to be the most important of the senses and the sense most closely allied with reason [32],” and hearing [30,31].
4. Simulation-based training has long been used in global health security and disaster medicine to adequately prepare the local health workforce for low frequency and/or theoretical events [33]. Medical simulation, which incorporates a case-based approached has also been used as part of predeparture preparation and to support training for healthcare providers and international settings. This strategy can be most readily applied to support the development of global burden of disease and clinical management competencies [34].
5. Twinning: Any innovation of a STEGH should incorporate a twinning or peer-mentorship approach. A twinning partnership between academic institutions in global health is an exchange in which all partners share knowledge and resources to work together towards mutually beneficial ends [35]. In addition to institutional partnerships, twinning is also beneficial between peers, and has been demonstrated among pairs of students from north and south institutions [36]. Twinning values the experience of all partners involved and emphasizes shared decision-making processes and sustainability of the partnership [35]. An example of a successful twinning approach in global health is the RAHI-STATHI Collaboration in India, which utilizes a “trainee-led” twining model for research and capacity strengthening initiatives between the University of Massachusetts Medical School and the Charutar Arogya Mandal medical college in western India [37]. One criticism of traditional STEGHs is that they are one-directional and can err on the part of benefitting the student while placing undue burden on the host organization [38]. A twinning approach helps to support the development of collaboration, partnering and communication skills, on both ends of the collaboration and may be one of the fewer strategies that helps bring to light considerations around social justice.

For students completing research and practice practicums, these approaches should be complementary to work-related tasks. In addition to virtual trainings, students should maintain regular communication and interaction with in-country collaborators and field teams to support the work and learn from local, contextual challenges and opportunities. Faculty, departments, and programs can support this by encouraging these connections and facilitating opportunities to create scopes of work, communication plans, and reporting plans.

Regular e-meetings give opportunities for students to practice their communication skills with people from different backgrounds and cultures. Similarly, these platforms provide opportunities for students from LMICs to interact with their counterparts and share research experiences; virtual approaches allow immersion to be bi-directional. In a remote applied learning experience, video conferencing platforms can provide the ability to video chat, share computer screens, and decipher vocal as well as visual cues and body language. This ongoing engagement provides students additional opportunities to contribute beyond a single project by offering strategic technologic support, thereby strengthening partner institutions’ capacities for remote communication and collaboration. The repeated and sustained interactions with peers are also beneficial to local students as they can develop a skillset to work effectively with people from different cultural backgrounds and start to develop professional networks for collaboration in the future [17].

### Addressing the Criticisms of STEGHS

There are many well-documented criticisms of STEGHs and the inequities of opportunities [39,40,41]. Before and during many STEGHs, international partners are expected to support coordination and facilitate logistics, particularly for those who are new to a setting. This can include organizing documents for visa applications, arranging drivers, organizing housing, developing agendas, and serving as a point of contact during emergencies. This can be time-consuming and sometimes un-remunerated work. While STEGHS vary in terms of length of stay, many have been criticized for being too short for students to contribute meaningfully [42]. Early weeks are spent acclimatizing to new culture, location, and workplace, giving little time to actively engage in work. An additional complaint of the short-term model is that it can be costly and time-inefficient for the organization to provide the necessary training and oversight to students who will only be working with them for a short time frame [42]. In fact, critics of short-term experiences have described them as potentially “one-directional and flow to the more powerful” party [38], benefiting the student more than the host organization.

Remote global health work can strengthen in equal measure north-south and south-north learning. Organized twinning experiences between students at global north and south institutions can further contribute to capacity strengthening (at both institutions) while also offering opportunities for cultural immersion on both sides [36,43]. In fact, remote global health work through twinning experiences can foster intercultural learning [44] not only through the acquirement of new knowledge at the cognitive level, but through sustained and engaged social interactions and multicultural group work. This type of work can also have logistical advantages; remote applied learning removes many of the coordination burdens from collaborators and instead provides opportunities for the team to focus on other aspects of work.

The current inability to travel could provide an opportunity to lengthen the time of the practicum and increase the overall duration during which students can engage with collaborators in a meaningful way, rather than spending a large proportion of their time acclimating to living in a new environment. In fact, this may provide a more grounded relationship where students and collaborators work together over time, testing the relationship, before eventually meeting in-country once travel bans are lifted. From the authors’ evaluation of the Johns Hopkins Center for Global Health training programs, many students wish they had spent more time preparing for their practicums in terms of technical skills, allowing them to be more productive upon arrival. This period of remote applied learning provides that preparation time, increasing the benefits for both students and collaborators.

It is also important to note the challenges and potential drawbacks of these virtual approaches. During this pandemic, many researchers around the world face an increase in work burden amidst competing priorities. Partners may not have the time to commit to providing mentored applied learning experiences, even remotely. Time zone differences, already a challenge in global health collaboration, may reduce opportunities for engagement. Further, if host institutions are closed, researchers and students may be responsible for their own information technology infrastructure at home which can carry unexpected and increased personal costs.

Communities also lose some of the indirect benefits from STEGHs including money spent on room and board and transportation services, which may be important sources of income.

## Conclusion

Supported, virtual, and well-mentored STEGHs can create new win-win dynamics for global health institutions in the global North and South. These opportunities may place a reduced burden on host institutions with students focused on giving support rather than receiving it. This serves to address an important ethical concern with STEGHs – trainee immersion and utilization of resources without truly giving back or providing services to the institution. Remote twinning opportunities can promote peer-to-peer collaboration and immersion, strengthening networks for junior researchers around the world. Finally, remote STEGHs may also better reflect professional relationships in global health, many of which are more remote.

We urge academic institutions to think critically about their STEGH programs and provide facilitated, innovative, well-supported, and partner-centric opportunities for students to continue applied learning from a distance.
